# Transcriptomic changes due to water deficit define a general soybean response and accession-specific pathways for drought avoidance

**DOI:** 10.1186/s12870-015-0422-8

**Published:** 2015-02-03

**Authors:** Jin Hee Shin, Justin N Vaughn, Hussein Abdel-Haleem, Carolina Chavarro, Brian Abernathy, Kyung Do Kim, Scott A Jackson, Zenglu Li

**Affiliations:** Center for Applied Genetic Technologies & Department of Crop and Soil Science, University of Georgia, Athens, GA 30602 USA

**Keywords:** Drought stress, Canopy-wilting, Glycine max, RNA-Sequencing, Quantitative trait loci (QTL), Genotype x environment

## Abstract

**Background:**

Among abiotic stresses, drought is the most common reducer of crop yields. The slow-wilting soybean genotype PI 416937 is somewhat robust to water deficit and has been used previously to map the trait in a bi-parental population. Since drought stress response is a complex biological process, whole genome transcriptome analysis was performed to obtain a deeper understanding of the drought response in soybean.

**Results:**

Contrasting data from PI 416937 and the cultivar ‘Benning’, we developed a classification system to identify genes that were either responding to water-deficit in both genotypes or that had a genotype x environment (*GxE*) response. In spite of very different wilting phenotypes, 90% of classifiable genes had either constant expression in both genotypes (33%) or very similar response profiles (*E* genes, 57%). By further classifying *E* genes based on expression profiles, we were able to discern the functional specificity of transcriptional responses at particular stages of water-deficit, noting both the well-known reduction in photosynthesis genes as well as the less understood up-regulation of the protein transport pathway. Two percent of classifiable genes had a well-defined *GxE* response, many of which are located within slow-wilting QTLs. We consider these strong candidates for possible causal genes underlying PI 416937’s unique drought avoidance strategy.

**Conclusions:**

There is a general and functionally significant transcriptional response to water deficit that involves not only known pathways, such as down-regulation of photosynthesis, but also up-regulation of protein transport and chromatin remodeling. Genes that show a genotypic difference are more likely to show an environmental response than genes that are constant between genotypes. In this study, at least five genes that clearly exhibited a genotype *x* environment response fell within known QTL and are very good candidates for further research into slow-wilting.

**Electronic supplementary material:**

The online version of this article (doi:10.1186/s12870-015-0422-8) contains supplementary material, which is available to authorized users.

## Background

Soybean is a primary contributor to worldwide food production. Water deficit dramatically limits growth and yield in crop plants, particularly for soybean, and the problem will likely be exacerbated by climate change. Irrigation is costly and often not a viable option for many soybean farmers. According to the USDA Economic Research Service report, only 8% of the U.S. soybean acreage is irrigated (http://www.ers.usda.gov/). Therefore, the development of drought-tolerant cultivars is critical in order to reduce the impact of drought stress on soybean production.

From a soybean breeding perspective, cultivar development is limited by the narrow diversity of elite germplasm, particularly with regard to drought tolerance [1]. Fortunately, a small number of land-races exhibit drought tolerance. One Japanese lace-race, PI 416937, retains yields in spite of drought [2] and was initially identified due to its slow-wilting phenotype. Further physiological characterization showed that PI 416937 has lower stomatal conductance [3], constant transpiration rate under vapor pressure deficit (VPD) above 2.0 kPa [4], and lower radiation use efficiency [5].

VPD is the difference between the water-vapor pressure in the air and the vapor pressure at which water-vapor condenses. At low VPD, dew forms, and, as VPD rises, plants transpire due to evaporation from the stomata. Interestingly, PI 46937 initially exhibits a conventional, linear increase in transpiration rate in response to VPD; yet, as the VPD continues to rise, the transpiration rate of PI 46937 stabilizes - a response that differentiates it from elite cultivars [4]. Transpiration rate is reduced within 40 minutes after exposure to cycloheximide, a bacterially-derived compound which inhibits protein translation [6]. This result indicates that symplastic/transcellular water pathway is maintained by continuous protein turnover. One explanation for PI 416937’s unique response to increased VPD is that the transcription of proteins mediating transpiration rate is being modulated relative to elite cultivars. To examine this possibility, we used deep sequencing of mRNAs (RNA-seq) to assay the transcriptomic response to water deficit in both PI 416937 and Benning, a common drought-sensitive cultivar.

Plant breeders are interested in identifying genes that confer drought-tolerance that can then be used for marker assisted selection. Since drought-tolerance is a highly complex trait, a whole-genome perspective is required. Still, previous attempts to understand drought tolerance using whole-genome transcript profiles often relied on the relative difference in pre- versus post-drought conditions for a single genotype [7]. Observing the final product of an elaborate chain of transcriptional events does not easily translate to either a better understanding of the plant’s responses or to improved plant varieties. One way to focus the search for useful drought tolerance genes is to compare differential expression of genes between genotypes that exhibit varying levels of drought tolerance. Indeed, this has been done previously in soybean for a relatively uncharacterized soybean variety [8]. While this study hinted genetic mechanisms that may confer drought resistance, the resistant variety used had not been extensively characterized in terms of its physiological response to water deficit, thus limiting the ability to connect genetic and physiological pathways. The study also illustrated the analytical difficulties of emphasizing only pairwise differences for samples that range across genotypes and environmental conditions. Here we apply a classification system to categorize genes based on the combination of genotypic and environmental response data. This approach allowed us to differentiate gene expression patterns that characterize a general soybean response from patterns that may be conferring PI 416937’s distinct transpiration rate profile. An additional benefit of comparing PI 416937 and Benning transcriptional profiles is that they are the parents for a mapping population previously used to identify slow-wilting QTL [9]; thus, genotypic differences in expression could be correlated to genetic polymorphisms segregating between the two lines.

## Results

### PI 416937 exhibits a slow-wilting phenotype

**Figure 1:**
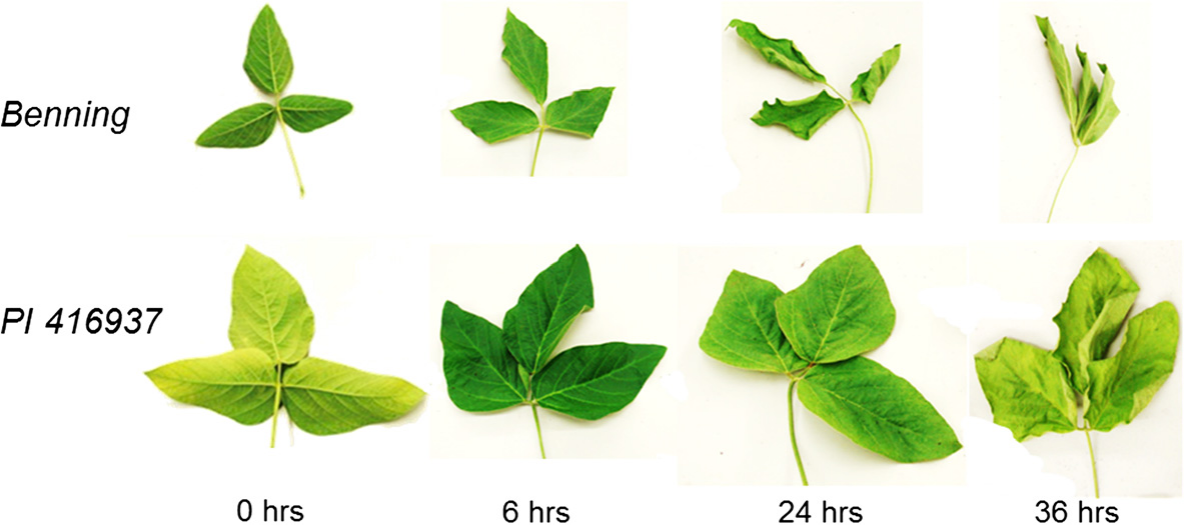
**Phenotypic response of Benning (sensitive) and PI 416937 (tolerant) soybeans after 0, 6, 24, and 36 hours of drying treatment.** Genotypes are shown as rows and time-points as columns. For 0 hr, leaflets at their widest point measured ~5 cm and ~7.5 cm for Benning and PI 416937, respectively.

As described in Methods, to create rapid water deficit, each genotype was gently removed from soil, washed, and exposed to constant ambient air for the remainder of the experiment. After 6 and 12 h of drying treatment, both genotypes did not show differences in wilting phenotype (Figure 1). However, the slow wilting genotype PI 416937 still maintained its shape whereas the fast wilting Benning was wrinkled and wilted after 24 hr of drying, clearly representing different levels of drought avoidance between two genotypes. After 36 hr, genotype PI 416937 also showed a wilting phenotype and Benning showed severe leaf curling.

### Transcriptome data for sensitive and tolerant soybean genotypes is highly reproducible

A total of 24 samples comprised of two soybean genotypes without drying treatment (controls, 0 h) and imposed drought stress (6, 12, and 24 hr) were used for transcriptome sequencing using Illumina HiSeq2000 system (Table 1). One library of PI 416937 6 hr replicate 3 was lost during library preparation procedure, thus PI 416937 replicate 2 was sequenced twice. Hiseq 2000 sequencing resulted in from 9.5 million (M) to 26.4 M reads per sample. The reads for each biological replicate were mapped independently to the reference genome. There were no genes with significant differences at the transcriptional level between PI 416937 6 hr replicate 2 analyzed in two different lanes, showing that the sequencing reaction and subsequent analysis introduced very little error (Additional file 1). Moreover, across biological replicates, the number of gene models with no significant difference ranged from 99.10% and 99.98% (Additional file 1), indicating high reproducibility.

**Table 1:** **Total read counts for treatments, genotypes, and replicates**

**Cultivar**	**Treatment**	**Bio Rep 1 reads**	**Bio Rep 2 reads**	**Bio Rep 3 reads**	**Total**
Benning	0	24.8 M	19.5 M	22.8 M	67.1 M
	6	25.4 M	18.1 M	18.7 M	62.2 M
	12	17.8 M	20.5 M	20.9 M	59.2 M
	24	21.0 M	26.5 M	15.4 M	62.9 M
PI 416937	0	22.6 M	20.5 M	26.4 M	69.5 M
	6	19.2 M	18.6 M	18.1 M^a^	54.1 M
	12	20.9 M	25.6 M	16.4 M	62.9 M
	24	15.4 M	21.6 M	12.7 M^b^	37.0 M

^a^PI 416937 was sequenced twice.

^b^PI 416937 Bio Rep 3 was an outlier relative to Rep1 and Rep2, thus excluded.

### PI 416937 and Benning have similar transcriptional response to water deficit but exhibit numerous genotypic differences

We attempted to combine data across genotypes and time-points in order to classify these expression profiles of expression into biologically relevant categories. Our categories were based on varying degrees of genotypic versus environmental responses (Table 2 and [10]). Generally, the classification system took into account the coefficient of variation across time-points as well as the statistical significance as assessed by *cuffdiff* (see Methods).

**Table 2:** **Expression types for all genes in the study**

**Type**	**Interpretation**	**Count**
*Untested*	Few transcripts present in any sample	19,391
*Low-expression*	Expression was too low to classify, but clearly present	8,488
*E-only*	Environmental response; gene expression levels change over the time-course, but there were no genotypic differences	9,208
*E-ambiguous*	Expression levels change over the time-course; genotypic differences may be present but minor.	3,619
*G-only*	No environmental response, but a constant genotypic difference across time-points	75
*GxE*	A substantial genotypic difference between two time-points; genotype is conditioning environmental response	542
*G + E*	An environmental response and a constant genotypic difference across time-points	84
*G + E- ambiguous*	Response is either a *GxE* or *G + E*, but difficult to specify which.	1,437
*Constant*	Expression was constant between genotypes and across time-points	7,290
*Ambiguous*	Expression was too erratic across replicates to classify.	3,511
Total		53,645

**Figure 2:**
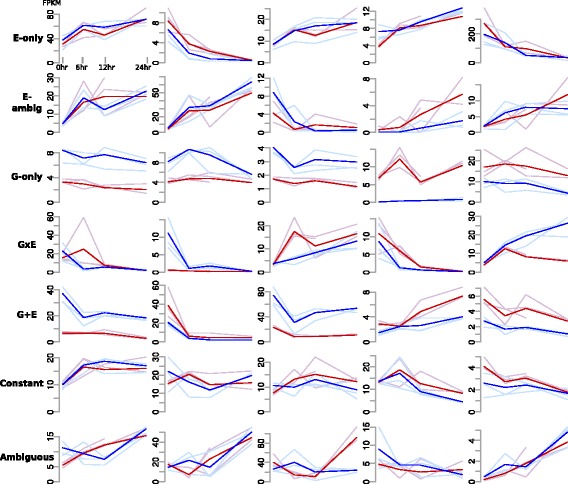
**Classification system for gene expression profiles.** Exhibited genes were randomly chosen from all genes within a category. Each row represents a single category. Blue and red colors indicate Benning and PI 416937, respectively. Light coloration indicates an individual replicate. Dark coloration indicates the mean profile across all replicates. Axis are labeled in the top right panel. Note, the scale of the y-axis differs for every plot.

Figure 2 illustrates gene expression profiles and their classification. *G-only* genes differed by genotype, but were relatively constant with regard to environmental change. *E-only* genes showed similar levels for both genotypes at individual time-points, but varied between time-points. *G + E* genes had both a genotypic difference and an environmental response. For *GxE* genes, the genetic background conditioned the environmental response. *GxE* genes had highly variable differences between the two genotypes at different time-points; for example, a *GxE* gene might have a log^2^ ratio FPKM_Benning_ to FPKM_PI-416937_ of 1.2 at 6 hr, but a difference of 3.3 at 12 hr. These *GxE* were particularly interesting because they suggest the genes that might be mediating phenotypic differences in wilting response. Because of the highly stringent criteria used to define the above categories, there were many cases where ambiguous gene expression profiles clearly exhibited a response, but were undefined. We further categorized these genes depending on whether they exhibited an extreme environmental or genotypic response for at least one time point. These are defined with the *ambiguous* suffix in Table 2 and Figure 2 (see Methods for more details).

A large fraction of gene models were not tested due to lack of transcript from sampled tissues and conditions (Table 2). Thirty-three percent of classifiable genes (all genes except *Untested, Low-expression*, and *Ambiguous*) were expressed at constant levels regardless of drought stress or genotype. Even with the large number of genes showing constant expression, very few exhibited a *G-only* response: 1%, [100 * (*G-only*/(*G-only* + *Constant*)]. Indeed, 96% of classifiable genes that were differentially expressed between genotypes – *G-only, GxE, G + E,* and *G + E-ambiguous* - exhibited an environmental response. Therefore, assuming the ratio of *GxE* to *G + E* genes holds for the *G + E-ambiguous* category, genotype generally appears to interact with the environment in a nonlinear way. All genes are listed along with their categories and expression profiles (Additional file 2).

### *E* gene profiles define a general soybean response

**Figure 3:**
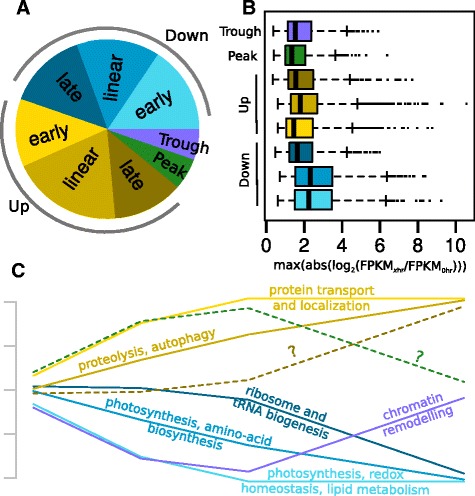
**General soybean transcriptional response to water deficit.** Color coding is consistent throughout the figure and defined in the pie chart. **(A)**, The distribution of E-type (E-only and E-ambiguous) genes are indicated as the proportion of the circle; n = 12,827. **(B)**, The maximum difference relative to 0 hr control of each gene is plotted with regard to its expression profile type. For each profile type, the mean, variance, and skewness of a distribution is estimated. Boxes indicate the middle quartile range of this distribution; lines indicate the highest and lowest quartile range. Dots indicate expression levels that extend beyond the estimated distribution. **(C)**, Expression profile models are illustrated, with functional enrichment categories labeling each profile.

Because we used two diverse soybean genotypes in this study, we could postulate a generic transcription response of soybean to water deficit. In order to elucidate this response, we further characterized the expression profiles of genes that showed a shared environmental response but little (*E-ambigous*) or no (*E-only*) genotypic difference (Figure 2), which we refer to as *E* genes. We formalized eight models to represent the average expression profile of these genes (Figure 3C): Up-early, in which genes were expressed to their maximum level within the first 6 hrs; Up-linear, in which genes continually increased over the time-course; and Up-late, in which genes stayed constant till the 24 hr time-point. We similarly defined a Down-early, Down-linear, and Down-late. Peak and Trough expression patterns were either up-then-down or down-then-up, respectively, across the time-course. Note that the shape of the expression profile, not its absolute level, dictates its classification.

The fraction of up and down-regulated genes was similar (Figure 3A). Roughly half of the up-regulated genes exhibited a linear increase in expression. In contrast, the down-regulated genes were more evenly divided between early and linear responses. We additionally assessed the maximum magnitude relative to the control (0 hr) of all *E*-genes. Most genes had a range of between 1 and 3 log_2_ units (2 to 8-fold greater or less than 0 hr), but some exhibited very high changes in expression, on the order of 6 to 8 log_2_ units (Figure 3B). While there was the expected correlation between set size and range, both linearly and late down-regulated genes appear to change more extensively than up-regulated genes with similar profiles. Thus, on balance, the number of transcripts in the leaf should decline with time under drought.

We assessed each profile set separately for possible enrichment in functionally related genes. Using AgriGO, we found distinct and highly significant patterns of functional bias (Table 3). Indeed, the fact that these categories are quite distinct indicates that our choice to group *E* genes by expression profiles was generally valid. Genes associated with photosynthesis and lipid metabolism were rapidly reduced and remain low (Figure 3C). A distinct set of photosynthesis genes were also continually reduced across the time-course. Towards 24 hr, genes involved in translation were down-regulated, resulting in a general decline in cellular metabolism. On the other hand, protein transport genes were up-regulated rapidly and stayed at relatively high levels. As cell metabolism declined, proteolysis and autophagy genes were increasingly transcribed. No significant categories were associated with Up-late genes. This observation stands to reason as most cellular processes appeared to decline in activity as water deficit continued. Somewhat surprisingly, Peak genes also show little or no functional enrichment. Interestingly, a clear drop in the transcription of chromatin remodeling genes was observed at 6 to 12 hr; transcription returned to 0 hr levels at 24 hr time point. Both Peak and Trough genes may represent genes that are oscillating in circadian cycles, and have little to do with drought response. Chromatin remodeling genes generally appear to be constant regardless of time of day [11], suggesting that this response is a reaction to initial water deficit and downstream physiological symptoms.

**Table 3:** **GO categories significantly associated with particular** ***E*** **-type expression profiles**

**Type**	**GO accession**	**Description**	**% in group**	**% BG** ^**b**^	**FDR**
Down-early (1400^a^)	GO:0015979	photosynthesis	3.29	0.63	1.8E-14
	GO:0055114	oxidation reduction	13.86	8.71	2.3E-07
	GO:0006629	lipid metabolic process	6.57	3.45	6.5E-06
	GO:0018130	heterocycle biosynthetic process	1.57	0.47	5.0E-04
	GO:0034641	cellular nitrogen compound metabolic process	4.36	2.30	5.9E-04
	GO:0045454	cell redox homeostasis	2.14	0.83	8.5E-04
Down-linear (1054)	GO:0015979	photosynthesis	3.51	0.63	3.0E-12
	GO:0006520	cellular amino acid metabolic process	4.08	1.60	2.5E-05
	GO:0043039	tRNA aminoacylation	1.52	0.35	3.0E-04
Down-late (1213)	GO:0006412	translation	20.86	3.21	4.5E-111
	GO:0034660	ncRNA metabolic process	3.30	0.85	9.0E-10
	GO:0044106	cellular amine metabolic process	4.04	1.66	3.9E-06
	GO:0015684	ferrous iron transport	0.74	0.12	2.2E-03
Up-early (952)	GO:0006886	intracellular protein transport	3.15	0.96	3.2E-05
Up-linear (1494)	GO:0044257	cellular protein catabolic process	2.61	0.61	7.3E-10
	GO:0016236	macroautophagy	0.60	0.05	4.7E-05
	GO:0015991	ATP hydrolysis coupled proton transport	1.07	0.21	5.4E-05
	GO:0007264	small GTPase mediated signal transduction	2.21	1.02	4.2E-03
Up-late (827)	No significant enrichment				
Peak (448)	No significant enrichment				
Trough (432)	GO:0006333	chromatin assembly or disassembly	2.23	0.37	1.6E-03

^a^Total number of genes within a category that have a GO annotation.

^b^Background model (BG) comprises all 29,641 soybean genes with a GO annotation.

We additionally assessed the GO enrichment of genes with very high-dynamic range in a category-wise fashion. These results generally bore out the functional enrichment analysis performed above, but were often less definitive (data not shown).

### Genotypic differences in transcription

Given the utility of characterizing *E* gene profiles, we extended this analysis to *GxE* genes. In this case we included a ninth model, Constant, in addition to those described above. No *E* genes should be constant, so the Constant model was not applied to that group, whereas one of the two genotypes of a *GxE* gene might show constant expression across time points.

**Figure 4:**
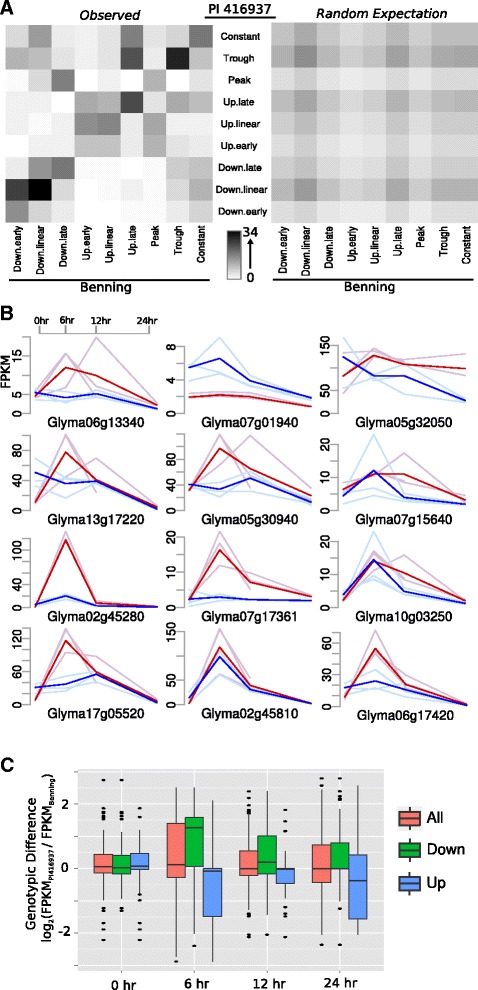
**Characteristics of response profiles of** ***GxE*** **genes. (A)**, Left panel shows a heat map reflecting the distribution of response profiles for all *GxE* genes in terms of their response in the two genotypes. The right panel shows the random expectation based on marginal frequencies of different profiles in the two genotypes. **(B)**, Twelve randomly sampled FPKM profiles for combinations of Peak and Down.late *GxE* profiles. Blue and red colors indicate Benning and PI 416937, respectively, as in Figure 2, where darker curves represent the mean of biological replicates shown in a lighter shade. **(C)**, Boxplots (as in Figure 3B) showing the genotypic difference at different timpoints for *GxE* genes that have the same response profiles, such as Up.late in Benning and Up.late in PI 416937. ‘All’ indicates both up and down-regulated genes while ‘Up’ and ‘Down’ indicate combined sets of up and down-regulated groups. The units of the y-axis are log_2_(FPKM_PI 416937_/FPKM_Benning_); positive values indicate that PI 416937 genes had higher expression than Benning at a given time-point.

We initially characterized the relative frequency for each possible combination of environmental responses specific to each *GxE* gene (Figure 4A). The pattern observed deviates strongly from random expectation (*p*-value < 10^−69^, Chi-squared test). As shown, most combinations fall along the linear axis, indicating that, even for *GxE* genes, the basic environmental response is the same, differing only by magnitude at a particular point. Indeed, there are very few examples of up-regulation in one genotype and down-regulation in another. In terms of combinations that are enriched but do not fall on the linear axis, most of these are not far from the axis, indicating that, even when expression profiles are distinct, they are not dramatically different. The most aberrant combination involves genes that are down-regulated late in Benning and show up-regulation and then down-regulation, or ‘peak’ profiles, in PI 416937. In examining the profiles of these genes, we found that PI 416937 genes most commonly peaked at a much higher levels than the relatively constant Benning genes. Note, this did not have to be true, as a gene could start higher in Benning than PI 416937 and then decline late as in Glyma07g01940 (Figure 4B); the absolute value of a profile is normalized by the maximum expression value, thus only the shape of the profiles are considered. Though the number was too small for robust enrichment statistics, of the seven genes that did show a sharp peak in early expression in PI 41937, such as Glyma17g05520 or Glyma07g17361, most are annotated as being transcription factors or as having some regulatory function at the protein level.

One hypothesis to explain PI 416937’s slow-wilting response is that genes associated with water transport in PI 416937 have reduced expression during water deficit, thus reducing transpiration and facilitating water retention (see Introduction). Only a very small fraction of *GxE* genes that were down regulated in PI 416937 had strikingly different expression profiles in Benning. It is possible that the functionally significant changes in gene expression are not qualitative, such as differences in profile, but quantitative, as suggested by the sharp diagonal in Figure 4A. Thus, given that most *GxE* genes exhibited similar profiles, we looked for time points that were commonly differentiating the two genotypes.

Looking only at genes that had the same profile – that fell along the diagonal in Figure 4A – we analyzed the genotypic differences for each gene at each time-point (Figure 4C). For example, because the units of the y-axis are log_2_(FPKM_PI 416937_/FPKM_Benning_), positive values indicate that PI 416937 genes had higher expression than Benning genes at a given time-point. We observed that no particular time-point had a biased genotypic difference when considering all profiles regardless of profile type (‘All’ in Figure 4C). When we grouped genes based on up or down-regulation, we observed a small bias at the 6 hr time-point; in other words, for genes that were similarly down-regulated in both genotypes, PI 416937 genes were not down-regulated as substantially as Benning, particularly at 6 hr. It is possible that these genes represent, in effect, a delayed response to water deficit. Whether this response is causally related to resistance to wilting, or is merely a byproduct of undergoing less water deficit is unknown. The lack of any visual phenotype at this stage would suggest the former (Figure 1). Still, this observation is the opposite of what would be expected under a model in which PI 416937 differentially down-regulates expression of a subset of genes in order to reduce transpiration levels.

### Genomic bias of *GxE* genes and known QTLs for slow canopy wilting

In our previous QTL study using 150 recombinant inbred lines (RILs) derived from a Benning and PI 416937 cross, seven QTL responsible for canopy wilting were identified. Of those, two and five favorable QTL alleles were found from Benning and PI 416937, respectively [9].

**Figure 5:**
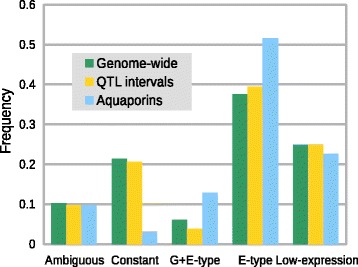
**Categorical distribution of genes across the genome (n = 34,178), within QTLs intervals previously identified (n = 755), and among aquaporins (n = 31).** Untested genes are not included in frequency calculation. Because the number of Aquaporins is small, all categories that showed a genotypic and environmental response – *GxE*, *G + E*, and *G + E*-*ambiguous* - were combined (*G + E*-type), as were categories that had an environmental response but no or small genotypic effects (*E*-only + *E*-*ambiguous* = *E*-type).

We compared the distributions of genes across the genome with those genes found within QTL intervals. There was no significant deviation from the expectation predicted by the genome-wide distribution (Figure 5). This finding is not surprising given that the QTL intervals are large and the majority of genes within a given interval are not expected to deviate sharply from the genome-wide distributions. Still, several genes within the known QTL have a clear *GxE* signal (Additional file 3 and Additional file 4) and are promising candidates for further investigation.

The distribution and/or expression levels of aquaporins are thought to be important in mediating PI 416937’s unique response to drought [4,6]. We additionally compared the categorical distribution of aquaporins to the genome-wide expectation (Figure 5). Though the sample is small, the distribution is significantly different than background (*p*-val < 0.05, Chi-squared test), indicating that aquaporins are more likely to respond transcriptionally to water deficit and also that they are more likely to have genotypic differences in their response. No aquaporin genes classified as being *GxE*-type genes fell within the known QTL interval.

## Discussion

Large-scale transcriptional reprogramming has long been interpreted as a mechanism of minimizing the effect of drought stress in plants [12,13]. The aim of this study was to identify a general response to drought stress in soybean and to compare differences at the transcriptional level between two accessions differing in canopy wilting phenotype. Although the drying treatment in this study is far from the actual drought stress under field conditions, it allowed us to measure transcriptional responses to water deficit, a major component of drought stress.

The majority of genes that we could confidently characterize a drought response were classified as *E* genes, indicating that they had roughly identical expression patterns for both genotypes (Figure 2 and Table 2). Prior to any noticeable phenotypic effect (Figure 1), dramatic transcriptional changes were occurring in both genotypes (Figure 2). While genes that are up or down-regulated late may be due to the physiological repercussions of canopy wilting, both early and linearly responsive genes are abundant (Figure 3) and likely responding to immediate water-deficit.

The most obvious response shared by sensitive and tolerant genotypes was down-regulation of photosynthesis related genes (Figure 3). There have been contradictory observations with regard to photosynthesis under drought stress, and this discrepancy is thought to be caused by differences in the severity of stress imposed on plants [14]. When plants encountered mild or moderate drought stress, photosynthetic acclimation was observed [12,15-17]. In contrast, photosynthesis has been reported as one of the primary process to be adversely affected under severe drought [16-19]. Thus, our treatment appears to be simulating severe drought.

Another response shared by sensitive and tolerant genotypes was up-regulation of genes associated with autophagy and nutrient starvation. Autophagy is an essential protein degradation process induced by abiotic stresses such as starvation, drought, salt, pathogen, and oxidative stress [20,21]. Photosynthetic constraint is one cause of carbon starvation, and carbon starvation induces autophagy [22]. The breakdown of oxidized proteins during oxidative stress and aggregated proteins in nutrient-starved cells can ensure cellular survival by maintaining cellular energy levels [23].

Prior to autophagy-related gene up-regulation, there was a rapid increase in genes involved in protein localization (Figure 3), primarily within the vesicular trafficking pathway. To our knowledge, this has not been observed in soybean, but has some precedent in *Arabidopsis* where up-regulation of related genes promoted osmotic stress tolerance [24]. Interestingly, other reports in *Arabidopsis* have implicated the downregulation of vesicle-trafficking-related SNARE protein in salt tolerence [25]; suppression of the gene in roots suppressed the production of reactive oxygen species by preventing vesicle fusion with the tonoplast. The connection between salt and water stress is complex [18], but the above findings in conjuction with those presented here, indicate that the shoot and root are exhibiting very distinct vesicle-trafficking profiles.

Chromatin remodeling genes have an unusual Trough expression pattern in both genotypes (Figure 3 and Table 3). Chromatin regulation responses to drought, cold, and salinity stress have been described in Arabidopsis [26,27]. It was reported that the histone H3 modification correlates with gene activation of the drought stress-inducible genes, such as responsive to dehydration (RD) 29A, RD29B, and related to AP2.4 (RAP2.4) [28]. Moreover some chromatin remodeling and modifying enzymes such as histone modification enzymes, linker histone H1, and components of chromatin remodeling complex have been shown to function in plant abiotic stress responses [27]. The initial down-regulation of these genes may reflect the expansion of euchromatin associated with the major transcriptional reprogramming that is occurring even at early stages of water-deficit, while the late up-regulation counters this trend, returning much of the genome to heterochromatin, under extreme physiological stress [29].

We had strong evidence for the differential expression between genotypes for 2,138 transcripts for at least one time-point (Table 2). For 25% of these, we could say with confidence that the genotype was conditioning the environmental response (*GxE* genes in Table 2). Less than 4% of these genotypically different genes had a constant expression in both genotypes (*G-only* genes in Table 2). Note, this result is not predicted by the ratio of *Constant* to *E-only* genes (Table 2), suggesting that genes that differ between genotypes are generally disposed to be stress responsive. This stands to reason in that stress-response regimes are likely to be selected under unique local environmental conditions [14].

The three major categories enriched in *GxE* genes were photosynthesis, innate immune response, and apoptosis genes, with a FDR of 5.2E^−06^, 2.3E^−07^, and 4.9E^−06^, respectively. Photosynthesis genes were substantially down-regulated under drought stress in both soybeans, however, photosynthesis genes of tolerant soybean were less affected at an early stage (6 hr) of water-deficit (Additional file 5). This is supported by prior studies that showed lower decrease of net photosynthesis rate or chlorophyll content in tolerant versus sensitive genotype under salt or drought stress [26,30].

Perhaps more interesting are the innate immune response and apoptosis genes, which show dramatic differences between genotypes and across conditions. Immune response genes are also a major target of local adaptation and have been previously identified as eQTLs for differential drought response [31]. Contrary to the expectation based on *E-only* profiles, apoptotic *GxE* genes are primarily down-regulated and vary most commonly in their initial expression levels (Additional file 6), indicating that physiological responses to wilting are not manifesting these differences. Still, the biochemical connection between water-deficit and apoptotic/immune response is tenuous, and functional enrichment in *GxE* categories may reflect overlapping local adaptations to stress in general, and not drought specifically. We anticipate that further fine-mapping studies will help resolve these questions.

To that end, one motivation for this study was the prior development of a genetic mapping population generated from a cross of the two lines assayed herein [9]. We did not identify a significant relationship between genes within previously identified QTL regions and *GxE* genes (Figure 5). Additionally, the region containing the strongest QTL, qSW-Gm12, with an R^2^ of 0.27 [9], did not have a significant enrichment in *GxE* or *G + E* genes (not shown). This result is not unexpected given that the QTL are not particularly well resolved and they could be mediating differences in slow-canopy wilting through any number of mechanisms [32,33]. Still, each of the QTL regions did contain *GxE* genes, and we propose these genes to be prime targets for fine-mapping, particularly those that have strikingly distinct expression profiles and act early in water deficit (Additional file 3 and Additional file 4).

The large majority of *GxE* genes exhibited quantitative differences in expression levels at particular points rather than qualitatively different profiles (Figure 4A). The exception to this trend was a small group of regulatory proteins that peaked in PI 416937 and remained relatively low and constant in Benning until 24 hr (Figure 4B). Though none of these genes fell directly within the range specified by the QTL mapping discussed above, chromosomes 5 and 17 contain QTLs nearby two of the most striking *GxE* profiles, Glyma02g45280 and Glyma17g05520. These genes are another set of promising leads in identifying solutions to problems posed by drought.

## Conclusions

Drought reduces yield in all crops, particularly soybeans. The response to drought is biochemically complex and entails major changes in gene expression. To that end, genome-wide expression data can be useful in improving plants to be robust to drought. However, it is difficult for plant researchers and breeders to employ genome-wide data because the results, in isolation, are often impressionistic and the experimental design does not focus on refining genomic loci that are causally underlying phenotypic variation. Here we used two relevant breeding lines, Benning and PI 416937 that have been used previously by our group as parents in a mapping population. These two lines exhibit strikingly different wilting responses, as shown here and in previous work, and their progeny were used to identify QTL underlying the slow-canopy wilting trait. We could therefore compare genes that have strikingly different profiles between genotypes with these QTL in order to resolve those QTL further and to understand their functions. To facilitate this comparison, we also developed a computational pipeline that allowed us to characterize the transcriptional response of each gene based on observations across the entire time-course and between the two genotypes. This approach allowed us to differentiate between genes that form a shared response and those that distinguish genotypes.

Taken together, we feel this study offers the following insights: 1) There is a general and functionally significant transcriptional response to water deficit that involves not only known pathways, such as down-regulation of photosynthesis, but also up-regulation of protein transport and chromatin remodeling; 2) Genes that show a genotypic difference are more likely to show an environmental response than genes that are constant between genotypes; 3) At least five genes that clearly exhibited a *GxE* response fell within the known QTL and are very good candidates for further research into slow-canopy wilting.

## Methods

### Plant materials and drought stress treatment

Both Benning (drought sensitive, elite US soybean cultivar) and PI 416937 (drought tolerant, Japanese landrace) were planted in the greenhouse on June 18, 2012 with 12/12 hours light/dark regime. At the R2 stage of flowering (September 7, 2012), plants were removed from pots, roots were washed, and the whole plants exposed to air. After 0, 6, 12, and 24 hr intervals, leaves were collected from both Benning and PI 416937 with three biological replicates, frozen in liquid nitrogen and stored at −80°.

### Total RNA extraction and library preparation

Tissues were ground under liquid nitrogen. The total RNA from leaf tissues was extracted using Trizol reagent (Invitrogen) and RNA-Seq libraries were prepared using TruSeq RNA Sample Prep Kits (Illumina) according to the manufacture’s recommendations. RNA-Seq libraries were constructed from two genotypes, four treatment time (0, 6, 12, and 24 hr), and three biological replicates. All libraries were barcoded using 24 index adapters, quantified using Bioanalyzer DNA 1000 Chip (Agilent Technology 2100 Bioanalyzer) and normalized to 10 nM.

### RNA sequencing and sequence analysis

All libraries were sequenced using the HiSeq2000 at the Genomics and Microarray Core at the University of Colorado Denver. Three lanes of HiSeq were used and each biological replicates was sequenced in different lanes according to proper blocking and randomization procedures [34]. Libraries were pooled equimolarly. Using TopHat2 (version 2.0.8b), sequencing reads (Table 1) were aligned to the Glyma 1.1 transcriptome (54,175 genes; 73,320 transcripts) determined by the Soybean Genome Project, US Department of Energy, Joint Genome Institute [35] (http://www.phytozome.net/soybean.php). Technical replicates (PI 416937 6 h replicate 2 and 3) were merged prior to testing the transcriptome variances. Mapped reads were provided as an input to Cufflinks (version 2.1.1) for transcript assembly, and then, differential expression of genes between genotypes and between treatments were analyzed by CuffDiff [36]. After consolidation of identical genes, 53,645 genes were included in the Cuffdiff analysis. Gene abundance is given as fragments per kilobase of exon per million fragments mapped (FPKM). Differential expression statistics were based on the log_2_ FPKM ratios [36].

### Classifier system for genotypic response profiles

All genes are classified via the following set of rules. For brevity, the following acronyms are used:

- *GD*, Genotypic difference at a single time-point calculated as log_2_ FPKM ratio of Benning genotype to PI 416937. In other words, a GD > 2 indicates a more than 4-fold difference in mean genotypic expression levels at a single time-point.
- *ED,* Environmental difference calculated as log_2_ FPKM ratio of 0 hr to 6, 12, or 24 hr for a given genotype. All genes have a 0 hr ED of 0.
- *SGD,* Significant GD with *q*-value <0.05.
- *SED,* Significant ED with *q*-value <0.05)
- *CV*^*+*^*,* 7 out of 8 time-points (4 time-points × 2 genotypes) had a coefficient of variation of <0.4 across the 3 replicates.
- *SS*^*+*^*,* Same sign for GDs; not a mixture of positive and negative GDs.

The classification key is described below as pseudo-code using underlined control words ‘IF’, ‘THEN’, and ‘ELSE IF’:

- **IF** No time-point in either genotype had an average FPKM of >1 **THEN** call *Untested*
- **ELSE IF** the highest FPKM of any time-point was <4 & the average FPKM across all time-points was <2 **THEN** call *Low-expression*
- **ELSE IF** CV^+^ & at least one SGD was >2.8-fold the GD at another time-point THEN call *GxE*
- **ELSE IF** CV^+^ & ≥3 SGDs & both genotypes have ≥1 SEDs & SS^+^**THEN** call *G + E*
- **ELSE IF** CV^+^ & ≤1 SGDs & both genotypes have ≥1 SEDs **THEN** call *E-only*
- **ELSE IF** CV^+^ & 0 SEDs for at least one genotype & ≥3 SGDs & SS^+^**THEN** call *G-only*
- **ELSE IF** CV^+^ & 0 SEDs for at least one genotype & ≤1 SGDs & no average ED >2 **THEN** call *Constant*
- **ELSE IF** At least one average ED >2 & ≥1 SGD had a *q*-value <0.001 **THEN** call *G + E-ambiguous*
- **ELSE IF** At least one average ED >2 **THEN** call *E-ambiguous*
- **ELSE** call *Ambiguous*

Of note, log scores are used in expression data because fold changes in expression are more meaningful than absolute changes. Yet, as commonly occurs in expression data, expression of one gene may be, for example, 0 at 0 hr and 50 at 6 hr. Though this is an important result with regard to our biological questions, it presents conceptual and, certainly mathematical, problems. For our purposes we used the following heuristic: if expression (FPKM) was <1 for one entry and >5 for the compared entry, then we gave the difference a score of 5, which represents an upper limit for the expression range across the time-course (Figure 3B). Alternatively, if the entry was <1 and the compared entry was <5, then we gave it a score of 0. An identical approach was taken for comparisons that would result in a negative score. For range calculations (Figures 3B and 4C), such values were ignored.

### Defining water-deficit response curves

The shape of expression profiles were characterized further. ED values in a time-course were normalized by the maximum ED. These profiles were then compared to nine explicit models, described graphically in Figure 3C. Explicit models are given as Additional file 7. The profile was classified as the model with the lowest sum of squared differences between model and observation for the given time-points.

### GO analysis

We use AgriGO web service to perform GO enrichment analysis on specific expression groups using Singular Enrichment Analysis [37]. Each analysis used ‘Glycine max v1.1’ as the species and ‘Soybean genome locus (phytozome v1.1)’ as the reference.

### Availability of supporting data

The raw data sets supporting the results of this article are available in the Short Read Archive under BioProject accession: PRJNA259941 (http://www.ncbi.nlm.nih.gov/bioproject/?term=PRJNA259941).

## Additional files

Additional file 1:
**Gene models with no significant difference at the transcriptional level between biological replicates.**
Additional file 2:
**An Excel spreadsheet containing all genes, their expression categories, and log**_**2**_ **ratios for genotypic difference and environmental responses.** 'Untested' genes are not included. Additional file 3:
**Profiles of** ***GxE*** **and** ***G+E*** **genes that overlap previously identified QTL.** See Figure 2 for details. Additional file 4:
**Genes that overlap previously identified QTL and exhibit** ***GxE*** **or** ***G+E*** **expression profiles.**
Additional file 5:
**Profiles of photosynthesis genes exhibiting** ***GxE*** **patterns.** See Figure 2 for details. Additional file 6:
**Profiles of apoptosis genes exhibiting** ***GxE*** **patterns.** See Figure 2 for details. Additional file 7:
**Explicit models used to characterize the expression profile of a particular gene.** A value of 1 represents the maximum expression level relative to control.

## Abbreviations

VPD: Vapor pressure deficit
QTL: Quantitative trait locus
RIL: Recombinant inbred line
FPKM: Fragments per kilobase of transcript per million mapped reads
