# Supplementary material for: Transcriptomic changes due to water deficit define a general soybean response and accession-specific pathways for drought avoidance
Source: BMC Plant Biol. 2015 Feb 3;15:26. doi: 10.1186/s12870-015-0422-8 (PMC4322458; doi:10.1186/s12870-015-0422-8)
Supplement: Additional file 1: — Gene models with no significant difference at the transcriptional level between biological replicates. [file 12870_2015_422_MOESM1_ESM.doc]

Additional File 1: Gene models with no significant difference at the transcriptional level between biological replicates

| Cultivar | Treatment | Rep1 vs. Rep2 | Rep1 vs. Rep3 | Rep2 vs. Rep3 |
| --- | --- | --- | --- | --- |
| Benning | 0h | 72949 (99.49) | 73215 (99.86) | 73232 (99.88) |
|  | 6h | 72941 (99.48) | 73231 (99.88) | 72770 (99.25) |
|  | 12h | 72849 (99.36) | 73284 (99.95) | 72660 (99.10) |
|  | 24h | 73279 (99.94) | 73063 (99.65) | 72967 (99.52) |
| PI416937 | 0h | 73038 (99.62) | 72738 (99.21) | 73169 (99.79) |
|  | 6h | 72857 (99.37) | 72853 (99.36) | 73320* (100.0) |
|  | 12h | 72797 (99.29) | 72636 (99.07) | 73296 (99.97) |
|  | 24h | 73305 (99.98) | - | - |

^*^ PI 416937 rep 2 was sequenced twice.
