# Supplementary figures and images for: Transcriptomic changes due to water deficit define a general soybean response and accession-specific pathways for drought avoidance
Source: BMC Plant Biol. 2015 Feb 3;15:26. doi: 10.1186/s12870-015-0422-8 (PMC4322458; doi:10.1186/s12870-015-0422-8)

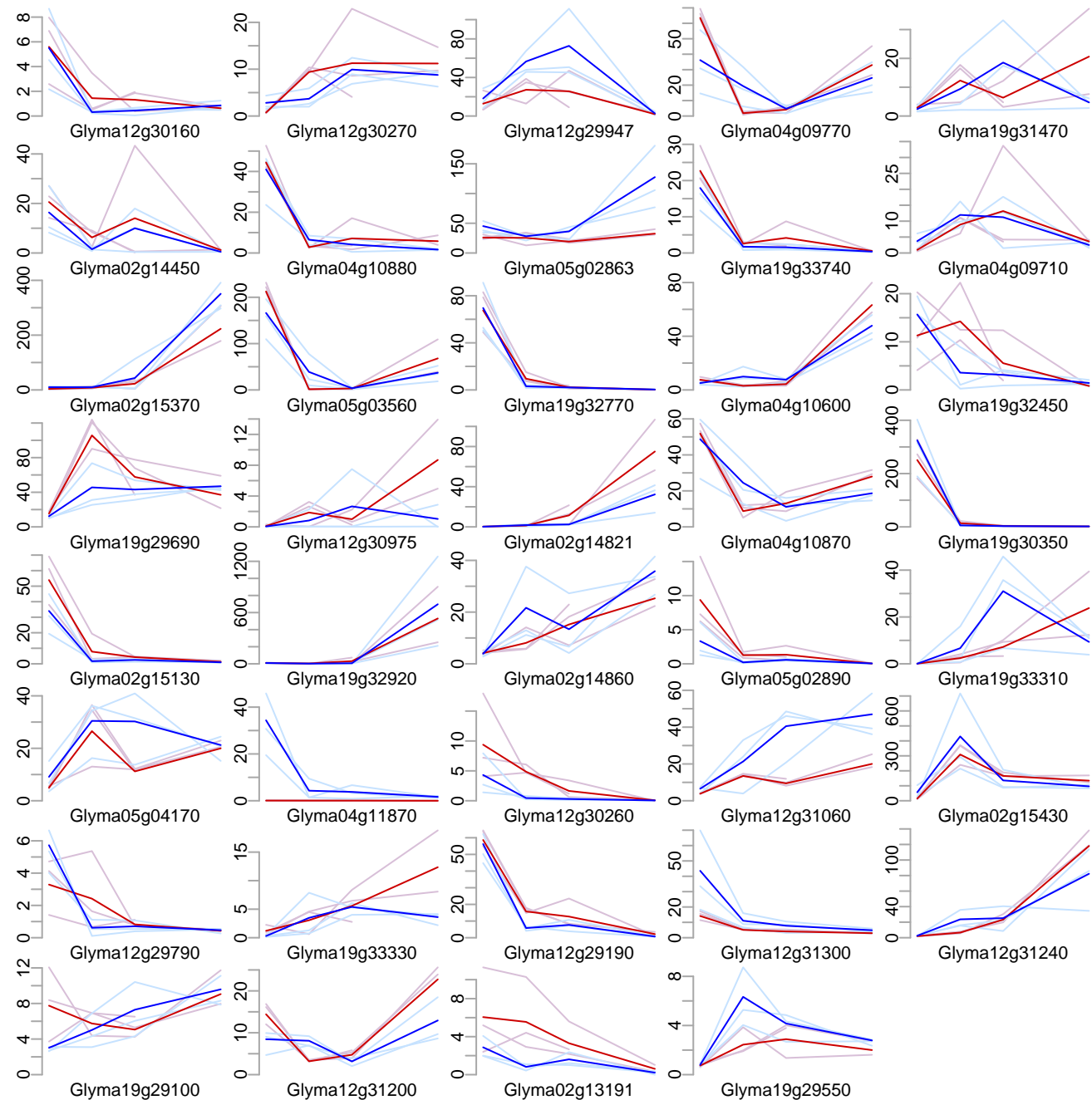

Supplement: Additional file 3: — Profiles of GxE and G+E genes that overlap previously identified QTL. See Figure 2 for details. [file 12870_2015_422_MOESM3_ESM.pdf]

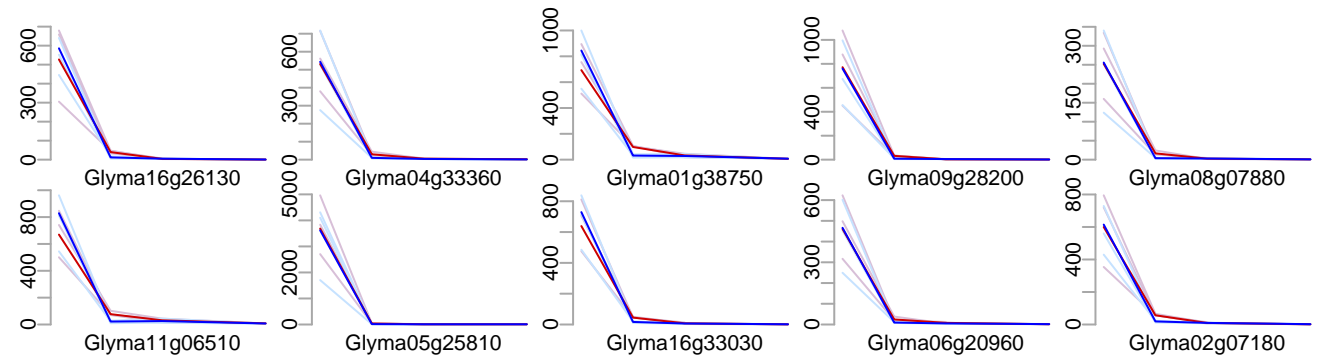

Supplement: Additional file 5: — Profiles of photosynthesis genes exhibiting GxE patterns. See Figure 2 for details. [file 12870_2015_422_MOESM5_ESM.pdf]

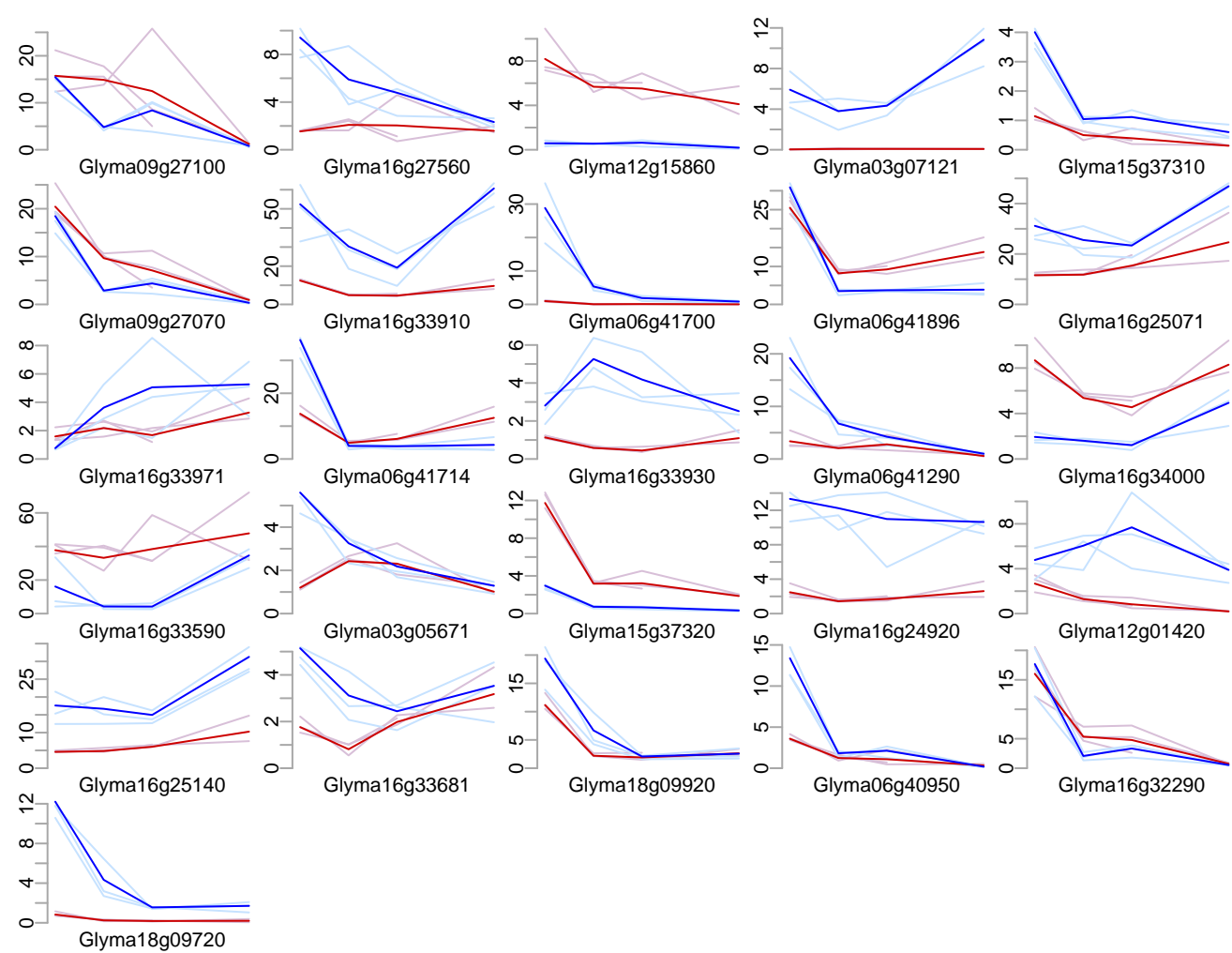

Supplement: Additional file 6: — Profiles of apoptosis genes exhibiting GxE patterns. See Figure 2 for details. [file 12870_2015_422_MOESM6_ESM.pdf]
