# Supplementary material for: Transcriptomic changes due to water deficit define a general soybean response and accession-specific pathways for drought avoidance
Source: BMC Plant Biol. 2015 Feb 3;15:26. doi: 10.1186/s12870-015-0422-8 (PMC4322458; doi:10.1186/s12870-015-0422-8)
Supplement: Additional file 4: — Genes that overlap previously identified QTL and exhibit GxE or G+E expression profiles. [file 12870_2015_422_MOESM4_ESM.doc]

**Additional File 6: Genes that overlap previously identified QTL and exhibit *GxE* or *G+E* expression profiles. Asterisk denotes genes that had early and strong *GxE* response based on visual inspection.**

| **Physical interval^a^** | **Gene interval^a^** | **Total Genes** | **Genes with G and E response** | **Type** |
| --- | --- | --- | --- | --- |
| Gm02:  11,076,739..  14,090,362 | Glyma02g12830-  Glyma02g15590 | 213 | Glyma02g13191 | G+E-ambiguous |
|  |  |  | Glyma02g14450 | G+E-ambiguous |
|  |  |  | Glyma02g14821 | GxE |
|  |  |  | Glyma02g14860 | G+E-ambiguous |
|  |  |  | Glyma02g15130 | G+E-ambiguous |
|  |  |  | Glyma02g15370 | G+E-ambiguous |
|  |  |  | Glyma02g15430 | G+E-ambiguous |
| Gm04:  7,830,812..  10,619,035 | Glyma04g09570-  Glyma04g11870 | 159 | Glyma04g09710 | G+E-ambiguous |
|  |  |  | Glyma04g09770 | G+E-ambiguous |
|  |  |  | Glyma04g10600 | GxE |
|  |  |  | Glyma04g10870 | G+E-ambiguous |
|  |  |  | Glyma04g10880 | G+E-ambiguous |
|  |  |  | Glyma04g11870* | G+E-ambiguous |
| Gm05:  1,800,376..  3,442,359 | Glyma05g02460-  Glyma05g04290 | 164 | Glyma05g02863 | GxE |
|  |  |  | Glyma05g02890 | G+E-ambiguous |
|  |  |  | Glyma05g03560 | G+E-ambiguous |
|  |  |  | Glyma05g04170* | G+E-ambiguous |
| Gm12:  32,496,908..  35,107,930 | Glyma12g29130-  Glyma12g31510 | 190 | Glyma12g29190 | G+E-ambiguous |
|  |  |  | Glyma12g29790 | G+E-ambiguous |
|  |  |  | Glyma12g29947* | G+E-ambiguous |
|  |  |  | Glyma12g30160 | G+E-ambiguous |
|  |  |  | Glyma12g30260 | G+E-ambiguous |
|  |  |  | Glyma12g30270 | G+E-ambiguous |
|  |  |  | Glyma12g30975 | G+E-ambiguous |
|  |  |  | Glyma12g31060 | G+E |
|  |  |  | Glyma12g31200 | GxE |
|  |  |  | Glyma12g31240 | G+E-ambiguous |
|  |  |  | Glyma12g31300 | G+E-ambiguous |
| Gm19:  36,557,287..  41,422,990 | Glyma19g28990-  Glyma19g33820 | 421 | Glyma19g29100 | GxE |
|  |  |  | Glyma19g29550 | G+E-ambiguous |
|  |  |  | Glyma19g29690* | G+E-ambiguous |
|  |  |  | Glyma19g30350 | GxE |
|  |  |  | Glyma19g31470 | G+E-ambiguous |
|  |  |  | Glyma19g32450 | G+E-ambiguous |
|  |  |  | Glyma19g32770 | GxE |
|  |  |  | Glyma19g32920 | G+E-ambiguous |
|  |  |  | Glyma19g33310* | G+E-ambiguous |
|  |  |  | Glyma19g33330 | G+E-ambiguous |
|  |  |  | Glyma19g33740 | G+E-ambiguous |

^a^Based on assembly version Glyma.Wm82.a1 (Gmax1.01)
