# Supplementary material for: Transcriptomic changes due to water deficit define a general soybean response and accession-specific pathways for drought avoidance
Source: BMC Plant Biol. 2015 Feb 3;15:26. doi: 10.1186/s12870-015-0422-8 (PMC4322458; doi:10.1186/s12870-015-0422-8)
Supplement: Additional file 7: — Explicit models used to characterize the expression profile of a particular gene. A value of 1 represents the maximum expression level relative to control. [file 12870_2015_422_MOESM7_ESM.doc]

Additional File 7: Explicit models used to characterize the expression profile of a particular gene. A value of 1 represents the maximum expression level relative to control.

| Profile | 0hr | 6hr | 12hr | 24hr |
| --- | --- | --- | --- | --- |
| Constant | 0 | 0 | 0 | 0 |
| Down.early | 0 | -1 | -1 | -1 |
| Down.linear | 0 | -0.33 | -0.67 | -1 |
| Down.late | 0 | 0 | 0 | -1 |
| Up.early | 0 | 1 | 1 | 1 |
| Up.linear | 0 | 0.33 | 0.67 | 1 |
| Up.late | 0 | 0 | 0 | 1 |
| Peak | 0 | 1 | 1 | 0 |
| Trough | 0 | -1 | -1 | 0 |
